# Dissociation in Patients with Non-Psychotic Mental Disorders

**DOI:** 10.11621/pir2021.0201

**Published:** 2021-06-30

**Authors:** Rinata Iskanderova, Valeriy V. Vasilyev

**Affiliations:** Izhevsk State Medical Academy, Izhevsk, Russia

**Keywords:** dissociation, dissociative phenomena, dissociative disorders, non-psychotic mental disorders, dissociation scale

## Abstract

**Background:**

Dissociation is a generally recognized phenomenon in psychology and psychiatry; however, questions are still not fully resolved about the difference between pathological and normal dissociation, as well as the role of dissociation, depending on its aetiology, in the formation of clinical manifestations of mental disorders.

**Objective:**

To complement the existing data about the significance of dissociation in non-psychotic mental disorders.

**Design:**

Using the Dissociative Experience Scale (DES), we screened 62 patients (13 male and 49 female) from the Non-Psychotic Conditions Inpatient Department of the Udmurt Republican Clinical Psychiatric Hospital (Izhevsk, Russia). Nineteen of the patients had mental disorders of organic aetiology and 43 patients had mental disorders of psychogenic aetiology.

**Results:**

Dissociation at the pathological level was detected in 12.9% of the patients, all of them female. Among patients with psychogenic disorders, the proportion of patients with pathological dissociation was more than three times that of patients with organic disorders. Among the particular dissociative phenomena, absorption had the highest average severity, both in the general sample and in each aetiological group of patients, while dissociative amnesia had the lowest average severity. The highest levels of dissociation were found in young female patients who had never been married. In patients with psychogenic disorders, the average dissociation severity was significantly higher than in the general population, while in patients with organic disorders it was significantly lower.

**Conclusion:**

The dissociation phenomenon may play a significant symptom-forming role in young women suffering from non-psychotic mental disorders of psychogenic aetiology. In the case of organic mental disorders, the severity of dissociative manifestations decreases even below the conditionally normal level, which may indirectly indicate the destruction of dissociative physiological mechanisms by an organic brain process.

## Introduction

Dissociation is a psychic phenomenon consisting in the detachment of certain experiences (usually painful ones) from a person’s consciousness. In a particular individual, this phenomenon may be manifest as the alienation of his own psychic acts or even his own “self”, the alienation of psychotraumatic events experienced by him, or the disintegration of memories of these events (McWilliams, 2011; Tarabrina, 2001). The term “dissociation” was introduced into practice in this specified meaning by the eminent French psychologist and psychiatrist P. Janet in the late 19th century (Janet, 2009). Dissociation is a common term in psychology and psychiatry, and it is considered an important mechanism in the formation of psychopathological symptoms. The role of dissociation in this process is recognized, in particular, by its inclusion in the “Dissociative disorders” block of modern psychiatric classifications, including the classification of mental and behavioral disorders in the current 10th revision of International Classification of Diseases (ICD-10). In the ICD-10 there are such forms of pathological dissociative disorders as dissociative amnesia, dissociative fugue, dissociative stupor, dissociative trance and possession disorder, dissociative motor disorder, dissociative convulsions, dissociative anaesthesia, Ganser syndrome (which is understood mainly as dissociative pseudodementia), and multiple personality disorder.

The predominant concept of the origin of dissociative disorders in the world today is the so-called traumatic concept, which considers these disorders a result of childhood mental trauma (Loewenstein, 2018; Reddy, Patil, Nayak, Chate, & Ansari, 2018). In this concept, great importance is attached to different types of child abuse — physical, emotional, or sexual (Dalenberg & Palesh, 2004; Dar & Hasan, 2018; Ross et al., 2008). Some authors even try to estimate the quantitative relationships between the intensity of childhood abuse and the risk of subsequent dissociative disorders (Granieri, Guglielmucci, Costanzo, Caretti, & Schimmenti, 2018; Schalinski et al., 2016). However, not all researchers view mental trauma as the main cause of dissociative disorders, and the nature of these disorders remains disputed (Canan & North, 2019). Moreover, there is a separate diagnostic category in the ICD-10, “Organic dissociative disorder”, which assumes the existence of a purely organic mechanism of the formation of dissociative disorders, unrelated to mental trauma. Finally, there are reports of an effect of dissociation on the course of a number of other neurotic and affective disorders, which may indirectly suggest involvement of the dissociation in the clinical manifestations of these disorders (Choi et al., 2017; Prasko et al., 2016).

Several authors note the existence of normal dissociation, which is part of a healthy psyche and is a mechanism of psychological defense, along with other similar mechanisms (Bokhan, Ovchinnikov, & Sultanova, 2017; McWilliams, 2011; Stepanova & Tokareva, 2017). The difference between normal dissociation and dissociation underlying mental disorders is understood in different ways by different researchers. Some view these two types of dissociation as qualitatively different phenomena, possibly even of different origins (Alayarian, 2019; Waller, Putnam, & Carlson, 1996). Others consider the differences between them to be purely quantitative, determined by the degree of adaptivity of dissociative manifestations at a certain level in a specific situation (Dell, 2002). Considering dissociation in this context and drawing data from Western researchers, Russian psychologist N.V. Tarabrina (2001) identified several particular dissociative phenomena that are components of a general dissociative continuum, extending along the norm–pathology axis. According to her study, the specified phenomena include: *absorption* — an affective absorbing of attention by a bright external object, up to complete mental fusion with it; *distraction* — uncontrolled shifting of attention from surrounding reality to internal experiences (“empty sight”, “waking dreams”); *depersonalization* — a feeling of one’s emotional change or loss of a sense of reality; *dissociative identity violation* — a transient or persistent experience of splitting or alienation of one’s “self ”; and *dissociative amnesia* — obscurity or total loss of memories about psycho-traumatic events.

Several scales have been developed to assess the severity of dissociative manifestations, including scales for large-scale epidemiological studies (Bernstein & Putnam, 1986; Carlson et al., 1993; Dalenberg & Carlson, 2010; Ross, Hebe, & Norton, 1989; Sanders, 1986). There are reports in the literature about studies of the prevalence and severity of dissociation in the general population, including cross-cultural aspects, that used these methods (Akyuz, Dogan, Sar, Yargic, & Tutkun, 1999; Ross, Joshi, & Currie, 1990; Xiao et al., 2006). The results of these studies show a rather high prevalence of dissociation not only among patients with mental disorders, but also among mentally healthy persons. At the same time, as mentioned above, the question of the differences between normal and pathological dissociation is still not fully resolved. Despite recognition of the significant role of dissociation in the clinical manifestations of various mental disorders (Friedl & Draijer, 2000; Gast, Rodewald, Nickel, & Emrich, 2001; Klaric & Lovric, 2018; Ross, Duffy, & Ellason, 2002), the relationship of dissociation to the aetiology of these disorders has also not been thoroughly studied. Identified knowledge gaps in understanding dissociation and its role in normal and pathological mental activity determine the relevance of this study.

## Methods

The objective of the study is to complement the existing data about the significance of the dissociation phenomenon in non-psychotic mental disorders.

Tasks of the study:

1. To determine the prevalence of pathological dissociation among patients with non-psychotic mental disorders;
2. To explore dissociation severity in non-psychotic mental disorders with different aetiology;
3. To explore dissociation severity in non-psychotic mental disorders at different demographic groups;
4. To compare dissociation severity in non-psychotic mental disorders of different aetiology with its severity in the general population.

The study was conducted with patients of the Inpatient Non-Psychotic Conditions Department of the Udmurt Republican Clinical Psychiatric Hospital (Izhevsk, Russia). All adult patients being treated in that department for the three-month period (from 1 October 2019 to 31 December 2019) who agreed to participate in the study were screened, for a total of 62 patients, 13 male and 49 female (gender balance 1 : 3.8). The age of the patients ranged from 21 to 74; the average was 50.4 ± 3.6 years. The diagnostic composition of the sample is presented in *Table 1*. Patients with non-psychotic mental disorders from two blocks of the ICD-10 — “Organic, including symptomatic, mental disorders” (F0) and “Neurotic, stress-related and somatoform disorders” (F4) — were included in the sample. Nineteen patients were diagnosed with a mental disorder from block F0, and 43 patients from block F4. In other words, the mental disorders of 19 patients had an organic aetiology and those of 43 patients had a psychogenic one.

**Table 1:** Diagnostic composition of the sample according to ICD-10

ICD-10 block	All patients	Male	Female
Organic, mental disorders including (Fsymptomatic, 0)	19	2	17
Neurotic, stress-related and somatoform disorders (F4)	43	11	32
Total	62	13	49

An experimental-psychological method was used to perform the study, the Dissociative Experience Scale (DES) developed by E.M. Bernstein and F.W. Putnam (1986). This scale is currently the most widely recognized tool in the world for identifying and measuring dissociative phenomena. We used the scale variant adapted by N.V. Tarabrina. The patients were screened for general severity of dissociation and its compliance with the normative level, which should not exceed 20.7 points according to the developers of the DES. Also, the severity of particular dissociative phenomena (absorption, distraction, depersonalization, identity violation, and dissociative amnesia) was estimated by focusing on separate paragraphs of the scale appropriate to the context of these phenomena. Student’s t-test was used to determine the statistical validity of the results.

## Results

Dissociation exceeding the normative level of the DES was detected in 8 patients (12.90% of the sample). All the patients with pathological severity of dissociation were female, and 16.33% of all female patients had a pathological level of dissociation. In patients with psychogenic disorders, dissociation at the pathological level was observed in 7 patients (16.28%), while in patients with organic mental disorders that level was found only in 1 patient (5.26%). Thus, among the patients with psychogenic disorders, the proportion of persons with pathological dissociation was more than three times that of the patients with organic disorders.

The average general dissociation severity in the sample according to the DES was 11.36 points (see *Table 2*). Among the particular dissociative phenomena, absorption, on average, was the most severe in the sample, followed (in descending order of average severity) by distraction, depersonalization, dissociative identity violation, and dissociative amnesia. It is particularly significant that the average level of dissociation in the patients with psychogenic disorders was almost twice that of the patients with organic mental disorders (p < 0.05). The average severity of each particular dissociative phenomenon in the former group was also higher.

**Table 2:** Average severity of general dissociation and particular dissociative phenomena

Dissociative phenomena	All patients	Patients with organic disorders	Patients with psychogenic disorders
General dissociation	11.36	7.83	13.02
Absorption	19.35	14.21	21.62
Distraction	15.20	11.18	16.97
Depersonalization	10.78	6.05	12.87
Identity violation	10.57	6.00	12.58
Dissociative amnesia	9.88	8.79	10.36

A comparison of general dissociation severity in patients by gender (see *Table 3*) shows that in women its average was slightly higher than in men (p < 0.05). The average severity of each dissociative phenomenon was also higher among female patients. In conjunction with the higher prevalence of pathological dissociation among the women, this result indicates that dissociative mechanisms in patients suffering from non-psychotic mental disorders are more developed specifically among the women.

**Table 3:** Comparison of the severity of general dissociation and particular dissociative phenomena in male and female patients

Dissociative phenomena	Male	Female	Significance of differences
General dissociation	10.79	11.57	p < 0.05
Absorption	18.46	19.59	p < 0.05
Distraction	13.84	15.56	p < 0.001
Depersonalization	10.38	10.89	p > 0.05
Identity violation	11.05	10.44	p > 0.05
Dissociative amnesia	7.91	10.40	p < 0.001

For studying dissociation severity in the different age groups of the patients, we used the World Health Organization’s (WHO) age periodization. The highest rate of general dissociation was observed in young patients (ages 18–44). In older age groups, that rate gradually decreased (see *Table 4*). The same pattern was observed for each particular dissociative phenomenon. The data suggests that dissociative psychological mechanisms in patients with non-psychotic mental disorders weaken with age.

**Table 4:** Average severity of general dissociation and particular dissociative phenomena in patients of diffferent ages

Dissociative phenomena	Young age (18–44 years)	Middle age (45–59 years)	Advanced age (60–74 years)
General dissociation	16.98	10.67	7.14
Absorption	25.74	22.77	10.86
Distraction	21.90	15.69	8.69
Depersonalization	17.56	8.40	6.46
Identity violation	16.30	8.95	6.59
Dissociative amnesia	12.51	10.08	7.32

An analysis of the dependence of dissociation severity on the marital status of the patients showed that the highest rates of both general dissociation and particular dissociative phenomena were observed in patients who had never been married, and the lowest rates were observed in widow and widowers (see *Table 5*). Married and divorced patients had medium values according to the considered indicators. It is likely that the described pattern is related to differences in the average age of the patients, as younger people are predominant in the “have never been married” group, while older people are predominant in the “widow and widowers” group.

**Table 5:** Average rate of general dissociation and particular dissociative phenomena in patients with different marital statuses

Dissociative phenomena	Unmarried	Married	Divorced	Widows widowers and
General dissociation	13.57	12.82	13.07	5.74
Absorption	26.67	22.16	26.67	4.61
Distraction	9.17	17.56	13.75	7.30
Depersonalization	10.00	12.33	11.87	6.25
Identity violation	11.87	12.26	13.34	3.87
Dissociative amnesia	14.05	10.15	10.95	6.70

The fact that both the severity of general dissociation and of particular dissociative phenomena appeared higher in divorced patients than in married ones, seems harder to explain, as patients’ ages in these categories did not differ much. It is possible that the level of stress in the divorced patients was higher due to dissatisfaction with their personal life or inherently greater emotional instability, which could have been one of the possible reasons for their divorce.

In this study, the average level of dissociation in the sample was also compared with the level of dissociation in the general population. The data about the level of dissociation in the general population were taken from the literature (Ross et al., 1990). We found that the average dissociation rate in the sample is significantly higher (p < 0.05) than in the general population (see *Table 6*). In patients with psychogenic disorders, the average dissociation compared to the corresponding value for the general population was even higher than in the study sample as a whole. This result allows us to hypothesize that in the case of psychogenic mental disorders, dissociative processes are indeed stronger and probably play a significant role in the formation of clinical manifestations of these disorders.

**Table 6:** Comparison of dissociation severity in the sample and in the general population

Cohort	Average severity of dissociation in the sample	Average of dissociation severity in general population (Ross et al., 1990)	Significance of differences
All patients	11.36		p < 0.05
Patients disorders with organic	7.83	10.80	p < 0.001
Patients disorders with psychogenic	13.02		p < 0.001

The average dissociation severity in patients suffering from organic mental disorders, by contrast, was significantly lower than in the general population. This leads us to conclude that in organic mental disorders, unlike mental disorders of psychogenic aetiology, dissociative processes in most cases do not intensify, but rather weaken.

## Discussion

The degree of scientific novelty of the results obtained is ambiguous. In particular, the results obtained in the present study, showing greater severity of dissociative processes in women suffering from non-psychotic mental disorders compared to men, confirm the data of several previous studies (Akyüz et al., 1999; Meyer & Waller, 1998; Olff, Langeland, Draijer, & Gersons, 2007; Vanderlinden, van Dyck, Vandereycken, & Vertommen, 1993). As a possible explanation of this fact, one study suggested the different nature of mental injuries that underlie dissociative disorders in men and women, in particular, the greater vulnerability of women to sexual violence (Wamser-Nanney & Cherry, 2018).

An interesting result is that dissociative processes in patients with non-psychotic mental disorders weaken with age. Several hypotheses can be suggested to explain this phenomenon. First, organic brain changes that occur with age, particularly of vascular or atrophic origin, may contribute to the weakening of dissociation (indeed, the results of this study indicate that organic brain damage resulted in less severe dissociation). Second, our results could be explained by the physiological age-related reduction of psychical mobility (Roshchina & Korsakova, 2020). Third, dissociation can decrease with age due to a certain stabilization of the individual’s living conditions and social relations (regular job, family, etc.), resulting in reduced stress and a “calming” of physiological drives. Finally, another possible explanation is gradual disactualization of childhood traumatic experience with age (if dissociation is considered from the point of view of the trauma concept). Clearly, all of these hypotheses require verification in future studies. Also, the present authors do not rule out the possibility that other explanations may be found.

An interpretation of possible causes for the influence of a person’s marital status on the severity of his or her dissociative reactions has already been given above. At the same time, it should be noted that the inverse relationship is also possible: Higher levels of average dissociation in persons who have never been married or in divorced persons may not be a consequence, but a cause of their dysfunctional marital status. This means that mental health problems related to the increased dissociation can lead to difficulties in starting a family or to issues in family relationships that might result in a divorce. Clarification of this question also requires further research.

The analysis of the severity of various particular dissociative phenomena in non-psychotic mental disorders showed that in the studied patients these phenomena appear unevenly. As already indicated, absorption reaches the highest level in the sample, followed by distraction, depersonalization, identity violation, and dissociative amnesia. Possibly this result is related to the different degrees of the adaptation violation connected with each of these phenomena and to the different degree of deviation of the corresponding mental functions. Obviously, such dissociative phenomena as absorption or distraction are less severe and disadapting than identity violation or dissociative amnesia. Apparently these dissociative phenomena, typically observed among mentally healthy people, constitute a certain level of so-called normal dissociation. These phenomena are amplified in non-psychotic mental disorders, so their severity becomes significantly higher. Such phenomena as identity violation or dissociative amnesia are not common in a healthy population; they occur, mainly, in cases of mental illness, and require more serious reasons for their formation. Probably they become intense only among a small percentage of non-psychotic patients with sufficiently severe mental disorders. Accordingly, the average rate of identity violation and dissociative amnesia in the sample is not so high in general. Depersonalization occupies a middle position among all the dissociative phenomena by the severity of its disadaptation and therefore its level in the sample is in the medium range.

An important finding of this study requiring a separate discussion is the weakening of dissociation in organic mental disorders that are accompanied by decreasing dissociative reactions even below the conditionally normal level. In our opinion this phenomenon may be caused by the destructive effect of an organic brain process on the physiological bases of dissociation, appearing to be a quite complicated and delicate mechanism in terms of brain physiology and requiring a sufficiently intact condition of its anatomical structures.

## Conclusion

In this study, dissociation at the pathological level was found in about one-eighth of all the patients with non-psychotic mental disorders. It can be assumed that dissociation plays a significant role in the formation of clinical manifestations of mental illnesses among those patients. Dissociation makes an especially major contribution in the cohort of young females who have never been married. According to the results obtained, the increase of dissociative mechanisms is characteristic mainly of mental disorders of psychogenic origin. In the case of organic mental disorders, on the contrary, these mechanisms weaken, possibly due to the destructive effect of the organic brain process on their physiological bases.

## Limitations

Generalization of the results of this study is limited by the small sample size.
